# Chondroitin sulfate-AuNRs electroactive scaffolds for on-demand release of biofactors

**DOI:** 10.1186/s12951-022-01261-8

**Published:** 2022-01-31

**Authors:** Maayan Malki, Assaf Shapira, Tal Dvir

**Affiliations:** 1 The Shmunis School of Biomedicine and Cancer Research, Faculty of Life Sciences, Tel Aviv University, 6997801 Tel Aviv, Israel; 2 The Center for Nanoscience and Nanotechnology, Tel Aviv University, 6997801 Tel Aviv, Israel; 3 Sagol Center for Regenerative Biotechnology, Tel Aviv University, 6997801 Tel Aviv, Israel; 4 Department of Biomedical Engineering, Faculty of Engineering, Tel Aviv University, 6997801 Tel Aviv, Israel

## Abstract

Controlled release systems are often integrated into polymeric scaffolds to supply essential biofactors to trigger physiological processes in engineered tissues. Here, we report the modification of chondroitin sulfate (CS) electroactive polymer with gold nanorods (AuNRs) to create hybrid macroporous scaffolds for enhanced on-demand release of growth factors and cytokines. The mechanical properties, porosity and degradation of the hybrid scaffolds were evaluated, and the viability and functionality of seeded cardiac cells were assessed. Following, the ability to control the release of the enzyme lysozyme, and the cytokine, stromal cell-derived factor 1 (SDF-1) by applying electrical stimulation, was demonstrated. The AuNRs were able to increase the current through the scaffolds, providing an efficient on–off release profile of SDF-1, which resulted in higher migration of cells expressing CXCR4 receptor. Finally, the engineered scaffolds were transplanted in rats and SDF-1 was released daily by electrical stimulation, promoting blood vessel-forming cell infiltration and vascularization. We envision that gold nanoparticles and other conducting nanomaterials can be incorporated into different electroactive materials to improve their capabilities not only for tissue engineering applications, but for a variety of biomedical applications, where enhanced electrical stimulation is needed.

**Graphical Abstract:**

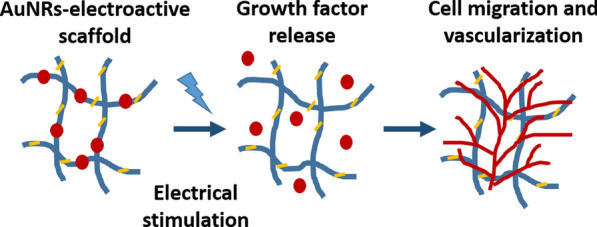

## Introduction

The field of tissue engineering aims to regenerate or repair lost or damaged tissues using biomaterial scaffolds, cells, growth factors (GFs) and other vital small biomolecules [1, 2]. Incorporation of GFs and cytokines into the scaffolding materials is essential for controlling many important physiological processes, including cell adhesion, proliferation, migration and angiogenesis [2]. Controlled release of such biofactors from the scaffolds allows to spatiotemporally regulate the formation of the engineered tissue. For example, controlling the release of angiogenic factors within a three-dimensional (3D) scaffold controls blood vessel formation within tissues in vitro and promotes endothelial cell (EC) infiltration and assembly into functional lumens in vivo [3, 4].

Several approaches have been utilized to incorporate GFs into 3D scaffolds [2] and release them into the cellular microenvironment, including chemical interaction of the factors into or onto the scaffold [3, 4], and their physical encapsulation within polymeric delivery systems [1, 5, 6]. Although the release of factors from such systems has triggered physiological processes, the release rate and profile were pre-determined by the synthesis process or according to the materials of choice, and no actual, on-demand control could be achieved. To better control the release of factors from biomaterials, stimuli-responsive polymers were employed. Different triggers were used to stimulate these materials and release the essential factors, including pH, temperature, exposure to an electric or magnetic field, ultrasound or light irradiation [7, 8]. Such materials have been used in a large variety of systemic treatments, including many common cancer therapies, but have shown limited success in tissue engineering applications. The regeneration process often requires long-term release, while most of the responsive systems only facilitate short release duration and irreversible responsive release [2, 9]. One of the commonly-used approaches for on-demand release of factors is based on passively loading a charged molecule into a polymer with the opposite charge to form electrostatic interactions, thereby trapping the desired molecule within the polymer. This has been achieved using different polymers, including chondroitin sulfate (CS), a naturally occurring polysaccharide with a negatively charged backbone [10]. In this example, the cation is loaded into the polymer backbone and upon the application of an electric field, the electrostatic bonds between the polymer and the loaded molecules break, releasing them to diffuse from the polymer matrix. Additional forces affecting this process are the local change in pH, which can lead to deswelling of the polymer and electrophoretic migration of the loaded cations towards the negative electrode. Recently we have demonstrated that using such an approach, controlled release of proteins such as lysozyme and stromal derived factor-1 (SDF-1) could be supplied to a developing tissue when electrical stimulation was applied [11]. We hypothesized that incorporation of conductive nanoelements within such electroactive polymers will increase the transfer of the electrical signal to trigger a more efficient release of GFs. Here, gold nanorods (AuNRs) were synthesized, characterized and incorporated into the backbone of the polymeric scaffolds. The hybrid scaffolds were then characterized in vitro for structure, mechanical properties, biocompatibility, degradation and for their ability to release biofactors. Then, the potential of the hybrid scaffolds to release SDF-1 in vivo and to trigger endothelial cell migration towards the scaffolds, infiltration and vascularization was assessed.

## Result and discussion

**Fig. 1:**
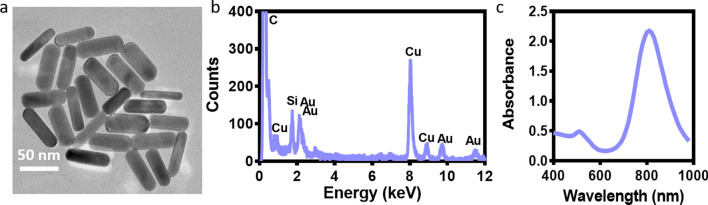
AuNRs characterization. **a** HRTEM micrographs of AuNRs (scale bar: 50 nm). **b** EDX analysis on HRTEM. **c** Vis–NIR spectrum of the AuNRs

AuNRs were synthesized using the seed-mediated growth method as we previously described [12] and characterized. High resolution transmission electron microscopy (HRTEM) was used to assess AuNRs size, as well as to verify the absence of aggregation. As shown, the rods were monodispersed with a length of 58 ± 5 nm and a diameter of 18 ± 3 nm (Fig. 1a). Energy dispersive X-Ray (EDX) analysis verified the presence of Au and its distribution on the HRTEM grid (Fig. 1b). Moreover, UV vis–NIR spectrum of the AuNR solution was examined to ensure that the sharp and strong absorption was centered at ~ 800 nm, indicating a homogenous size distribution (Fig. 1c).

**Fig. 2:**
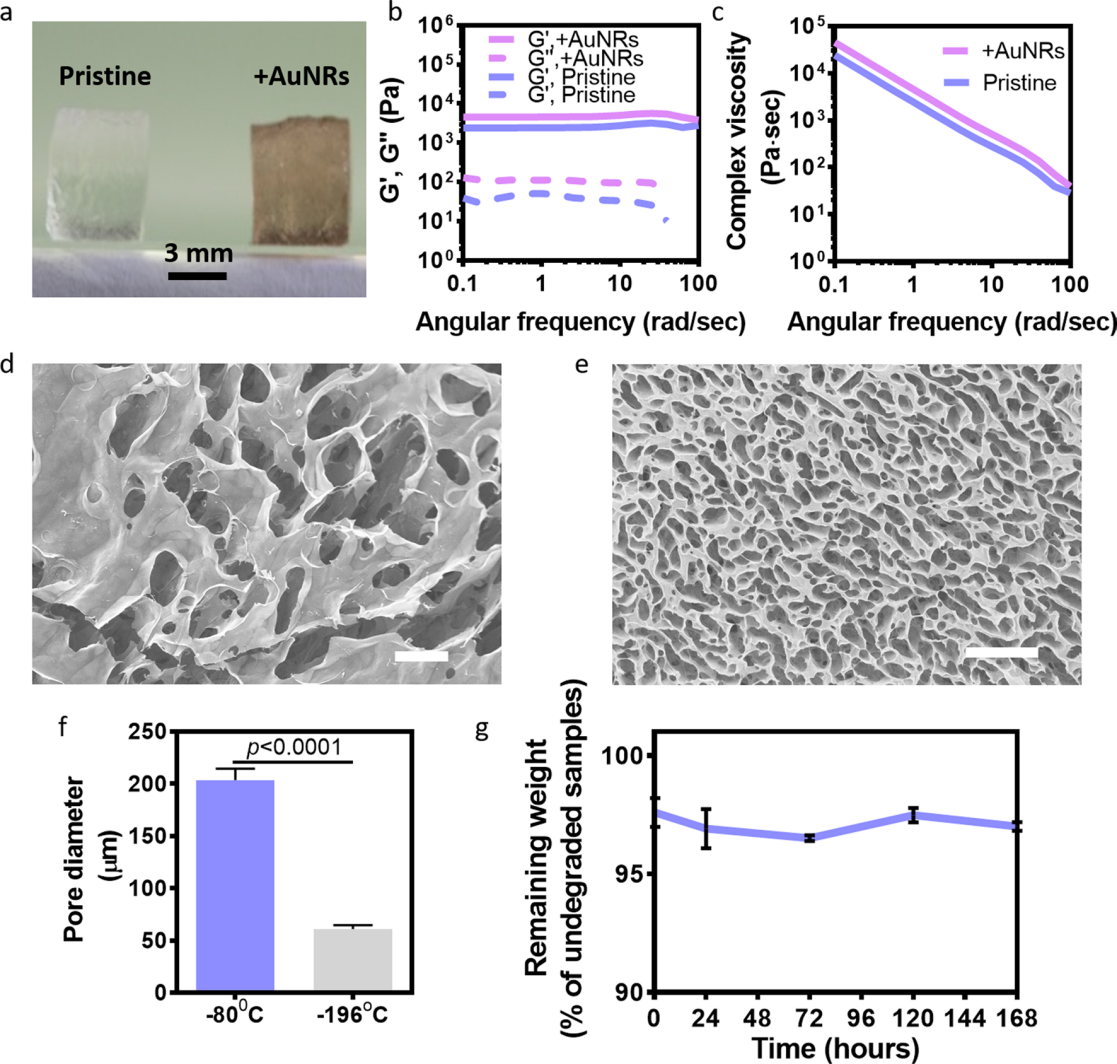
CS-AuNRs macroporous scaffolds. **a** The crosslinked hydrogel prior to lyophilization. **b**, **c** Rheological properties of the hydrogel. **b** Loss and the storage moduli plotted versus the angular frequency. **c** Complex viscosity plotted versus the angular frequency. **d** SEM image of the macroporous scaffold after freezing at -80 °C. **e** SEM image of the macroporous scaffold after freezing at - 196 °C. Scale bar = 200 µm. **f** Mean pore diameter of the lyophilized scaffolds. **g** Degradation of the scaffolds during incubation in collagenase solution at 37 °C

To incorporate the AuNRs into the backbone of the CS hydrogel, AuNRs solution was added at the last step of gel formation, resulting in a red-wine-colored transparent hydrogel (Fig. 2a). To quantitatively evaluate the mechanical properties of the CS-AuNRs hydrogel, the mechanical response of the samples upon deformation under periodic strain was measured. The obtained data for the pristine and the CS-AuNRs hydrogels were characterized by the storage modulus G′, which exhibited a pronounced plateau in the frequency range investigated, and by the loss modulus G′′, which was considerably lower (Fig. 2b). Although the loss and storage moduli curves were parallel in each frequency, a slight increase of the representative curves of storage and loss moduli of the CS hydrogel over the CS-AuNR hydrogel was noticed. These results indicated that the CS-AuNRs hydrogel was slightly stronger than the pristine CS hydrogel, probably since the rods were serving as space fillers [13]. Plotting the complex viscosity versus angular frequency showed that the complex modulus decreased as the frequency increased. Moreover, the complex viscosity of the CS-AuNRs hydrogel was slightly higher than that of the pristine hydrogel (Fig. 2c).

We next sought to fabricate macroporous scaffolds from the hydrogels. Porous structure is essential in tissue engineering to accommodate a large number of cells, for the formation of homogenous and continuous tissues and for efficient cell penetration and mass transfer. Pore size and structure of the scaffolds can be tailored by applying different freezing regimes, followed by lyophilization [14]. Here, the scaffolds were crosslinked by 21% EGDGE, frozen at – 80 or − 196 °C, lyophilized, imaged by scanning electron microscopy (SEM) and the pores were analyzed. During the slower freezing step at − 80 °C, larger ice crystals were formed. After lyophilization, these crystals left larger pores as compared to the snap freeze at − 196 °C (Fig. 2d–f). In order to examine the stability of the CS-AuNRs scaffolds, a degradation test was performed. To imitate in vivo degradation, the lyophilized scaffolds were incubated in a collagenase solution at 37 °C for a week, and their dry weight was measured at several time points. As shown, only a slight degradation (3%) was detected after 7 days with the enzyme (Fig. 2g).

**Fig. 3:**
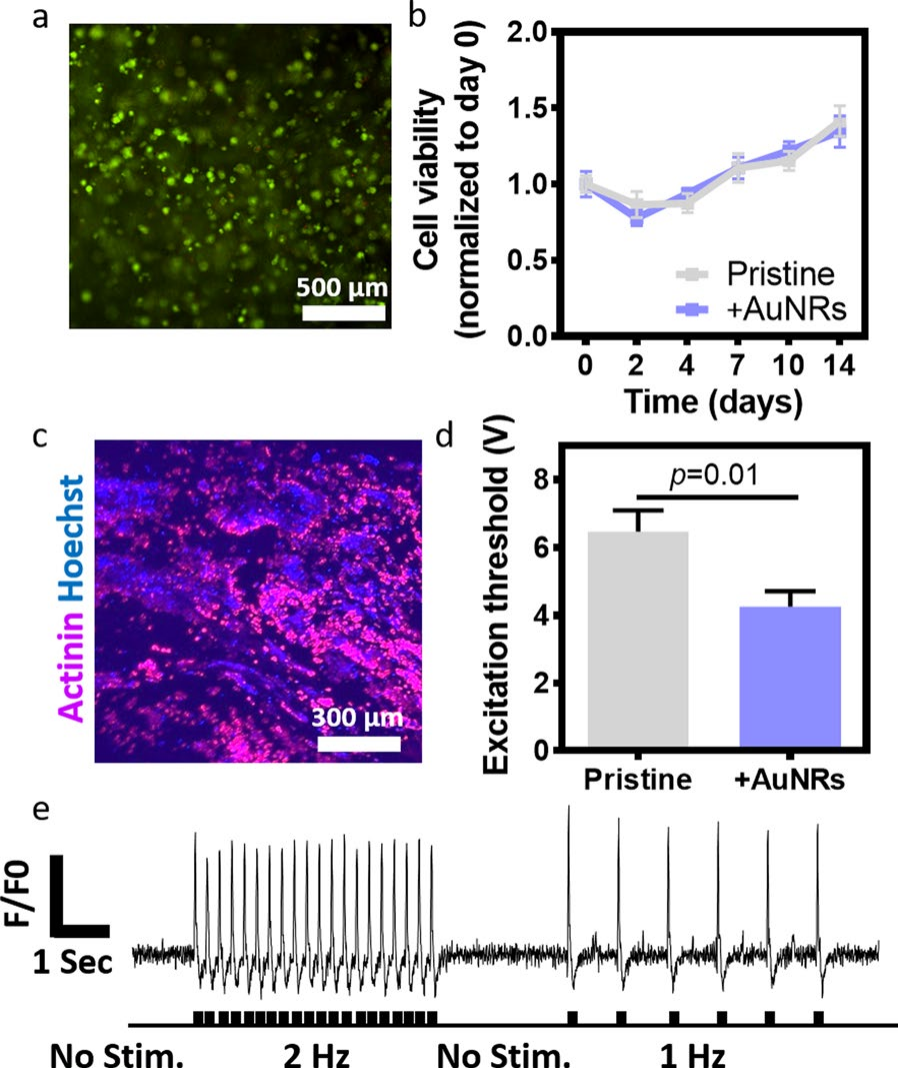
Cardiac cells viability and function within the CS-AuNRs scaffolds. **a** Cardiac cell viability as indicated by live/dead staining. **b** Cardiac cell viability within the scaffolds. **c** Immunofluorescence staining of the engineered tissue within the CS-AuNR patch. The construct was stained for cardiac α-sarcomeric actinin (pink) and cell nuclei (blue). **d** Excitation threshold of the engineered tissues. **e** Quantification of calcium flux (via normalized fluorescence intensity) without electrical stimulation or with 1- or 2-Hz stimulation. The stimulation pattern is shown at the bottom

We next sought to assess the biocompatibility of the hybrid scaffolds and their effect on cardiac tissue function. Here, we chose to use the scaffolds with the smaller pore size to accommodate cardiac cell clusters of up to 100 µm. Cardiac cells were isolated from the left ventricles of neonatal rat hearts and seeded within the pristine or AuNRs-modified scaffolds. The cells were incubated for up to 14 days and cell viability in the presence or absence of AuNRs was evaluated by live/dead and Presto Blue assays. As shown, the scaffolds were able to maintain cell viability (Fig. 3a). The isolated cells were composed of a mixed population of cardiac muscle cells, which do not divide, and cardiac fibroblast, which have a proliferative capability. Monitoring cell proliferation over 14 days further demonstrated a slight increase in cell population on both the pristine and hybrid materials (Fig. 3b), indicating the biocompatibility of the AuNRs. Immunostaining of the cell constructs for cardiac muscle markers revealed that most of the cells expressed cardiac sarcomeric actinin, indicating that the AuNRs scaffolds did not affect the more sensitive muscle cells (Fig. 3c).

Our group has recently shown the ability of gold nanowires and nanospheres to improve cardiac electrical signal throughout macroporous scaffolds, electrospun fibers and hydrogels [13, 15–17]. Here, we sought to evaluate the potential of the nanorods to improve the transfer of the signal in the scaffolds made of chondroitin sulfate. Therefore, we next, investigated the functionality of the assembled tissues as indicated by the ability to generate synchronous contractions throughout the tissue. Cardiac constructs were subjected to an increasing external electrical fields, and the minimum voltage needed to induce synchronous contractions of the entire tissue at the defined frequency was defined as the excitation threshold. As shown, cardiac cells grown in the CS-AuNRs modified scaffolds reacted to significantly lower electrical fields compared to those grown on the pristine scaffolds (Fig. 3d). To evaluate the electrical coupling of the extracellular signals, calcium imaging was performed. Electrical stimuli were transiently applied at a frequency of 1 or 2 Hz and the corresponding calcium wave fronts were recorded (Fig. 3e). Overall, these results indicated that the CS-AuNRs are biocompatible and can significantly contribute to tissue formation and its function.

**Fig. 4:**
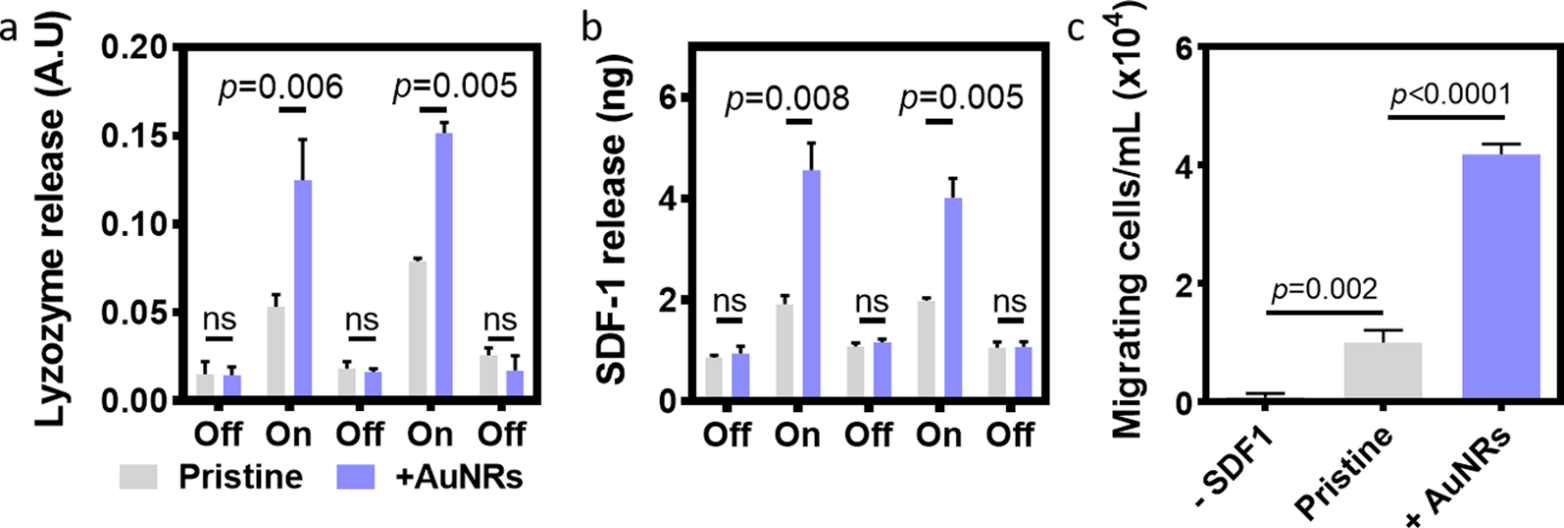
Controlled release of biofactors from the CS-AuNRs scaffolds. **a** Release of lysozyme. **b** Release of SDF-1. **c** Cell migration induced by SDF-1 released from pristine or AuNRs-modified scaffolds

We have recently shown the ability to use CS as an electroactive acellular material to control the release of proteins and growth factors [11]. Here, we sought to improve the control over the released factors by enhancing the electrical signal through the AuNRs within the scaffolds. Lysozyme was first selected as a model protein. The protein was introduced to the scaffolds and allowed to electrostatically bind the sulfate groups. Alternating the electrical stimulation broke the electrostatic interactions, demonstrating an on/off release profile of the protein from the pristine and AuNRs scaffolds, with significantly higher release within the modified scaffolds (Fig. 4a). Since the conducting nanorods serve as nano antennas, the applied electrical signal was transferred more efficiently within the CS-AuNRs scaffolds, promoting higher release of the factor. It is important to note that during the “off” cycle, no significant release could be detected between the groups.

In order evaluate the ability of the system to release physiologically relevant factors, we next focused on the release of SDF-1. This cytokine is a stem cell chemoattractant that triggers the migration of CXCR4 expressing cells, such as adult stem cells and endothelial progenitor cells, towards its source [18], promoting angiogenesis [1]. SDF-1 was loaded and stored in the pristine and CS-AuNRs scaffolds by electrostatic interactions, and was released upon application of an electric field. Similar to the lysozyme, an on/off release profile could be obtained by alternating the electrical field (Fig. 4b). Furthermore, the released cytokine from the CS-AuNRs scaffolds was able to recruit significantly higher number of CXCR4-positive cells compared to the released factor from pristine scaffolds or from CS-AuNRs scaffolds without SDF-1 (Fig. 4c).

**Fig. 5:**
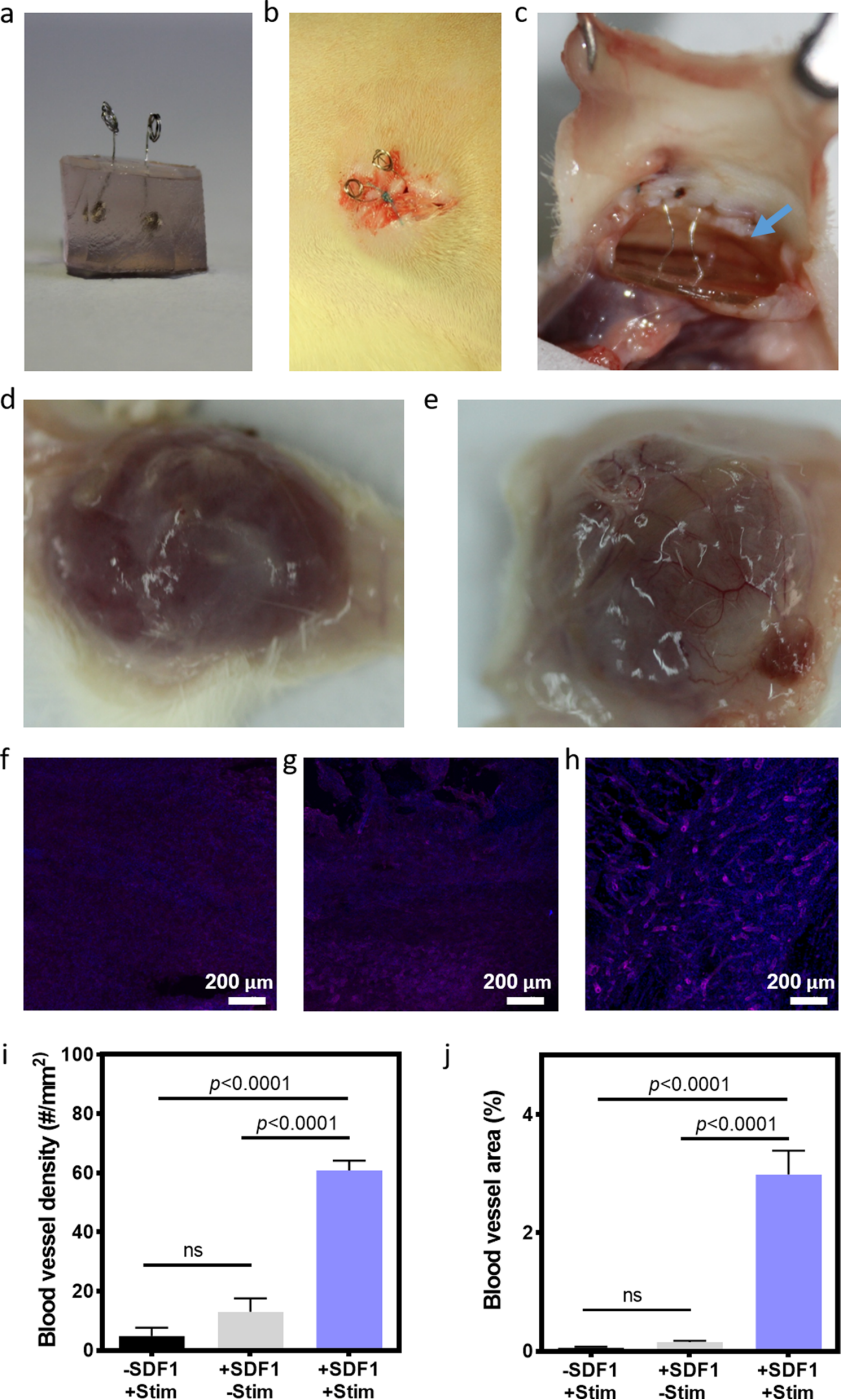
In vivo vascularization after release of SDF-1. **a** Macroscopic side view of the CS-AuNRs before implantation. **b** CS-AuNRs scaffold after subcutaneous implantation. **c** Macroscopic side view of the CS-AuNRs scaffold 8 days after transplantation in rats. **d** Macroscopic examination of the CS-AuNRs scaffolds loaded with SDF-1 without electrical stimulation. **e** Macroscopic examination of the CS-AuNRs scaffolds loaded with SDF-1 after 2 min/day stimulation for 8 days. **f**–**h** Immunostaining of sections of the transplanted scaffolds for smooth muscle actin. **f** Control scaffold loaded with BSA and stimulation. **g** Scaffold loaded with SDF-1 without stimulation. **h** Scaffold loaded with SDF-1 and electrically stimulated. **i**, **j** Quantification of blood vessels. **i** Blood vessel density. **j** Blood vessel area

Finally, to prove the potential of the CS-AuNRs scaffolds to release functional biofactors in vivo, two platinum wires were introduced into the scaffolds at the hydrogel preparation stage before lyophilization (Fig. 5a). The scaffolds were subcutaneously transplanted into rats, with the platinum electrodes projecting from the animals in order to enable application of electrical stimulation (Fig. 5b). Here, 3 groups were tested, including CS-AuNRs scaffolds loaded with SDF-1 and application of an electrical field, CS-AuNRs scaffolds with SDF-1 without electrical field, and CS-AuNRs scaffolds loaded with bovine serum albumin (BSA) instead of SDF-1 and application of electrical field. The scaffolds were connected via the electrodes to a stimulus generator and a voltage of 2 V was applied for 2 min once a day. Eight days after transplantation the rats were sacrificed and the scaffolds and the surrounding tissue were extracted (Fig. 5c). While no significant vascularization was observed by macroscopic examination of the scaffolds containing SDF-1 but without electrical stimulation (Fig. 5d), the electrically stimulated SDF-1-loaded scaffolds were filled with blood vessels (Fig. 5e). Following, the extracted implants were fixed and immune-stained for smooth muscle actin, indicating on matured blood vessels. Confocal microscopy of the explanted scaffolds revealed significant differences in the formation of mature blood vessels. While low number of blood vessels were identified in both control groups, the CS-AuNRs implants loaded with SDF-1 and electrically stimulated exhibited a significantly higher number of blood vessels (Fig. 5f-h). Quantification of the blood vessels within the constructs revealed a significantly higher number of vessels per square millimeter of tissue in the CS-AuNRs scaffolds that were loaded with SDF-1 and electrically stimulated (60.87 ± 3.23), compared to the control groups (4.81 ± 1.42 without SDF-1 and 13.04 ± 4.57 without stimulation; Fig. 5i). Moreover, Fig. 5j revealed that the percentage of the total area of blood vessels within the stimulation + SDF-1 group was significantly higher (~ 3%), compared to the group without SDF-1 (~ 0.05%), or with SDF-1 but without stimulation (~ 0.15%). Taken together, these results indicate the ability of the scaffolds to control the release of SDF-1 following electrical stimulation in vivo, leading to improved blood vessel infiltration into the scaffold.

## Summary and conclusions

Electroactive polymers such as chondroitin sulfate are essential materials for controlled release of biofactors in tissue engineering and for other applications. Here, we incorporated AuNRs into chondroitin sulfate electroactive hydrogels. The hybrid hydrogels were then lyophilized to generate CS-AuNRs-modified macroporous scaffolds. The scaffolds were characterized and their potential to maintain cell viability, support cell proliferation and enhance the electrical signal between cardiac cells was demonstrated. Furthermore, the ability to improve the release of growth factors and cytokines by incorporating AuNRs into the backbone of the chondroitin sulfate macroporous scaffolds was shown in vitro. Finally, we have demonstrated the ability of the CS-AuNRs scaffolds to release angiogenic factors in vivo and promote vascularization. We envision that gold nanoparticles and other conducting nanomaterials can be incorporated into different electroactive materials to improve their capabilities not only for tissue engineering but for a variety of biomedical applications, where enhanced electrical stimulation is needed. These may include drug delivery systems, pacemakers, electroporation, etc.

## Materials and methods

### AuNRs synthesis

AuNRs were synthesized as previously described [12]. Briefly, Cetyl Trimethyl Ammonium Bromide (CTAB) solution (15.3 mL, 0.20 M) was mixed with 1.7 mL of 1.016 mM HAuCl_4_. The solution was separated into two parts, seed and growth (2 mL and 16 mL, respectively). A 0.12 mL of ice-cold 0.01 M NaBH_4_ was added to the seed solution, resulting in the formation of a brownish-yellow solution. The seed solution was vigorously stirred for 3 min followed by a 1-h incubation at 25 °C to ensure total decomposition of the borohydride. In parallel, 0.15 mL of ascorbic acid (0.115 M) and 0.15 mL of AgNO_3_ (20 mM) were added to the growth solution. The seed solution was diluted in distilled deionized water (DDW) at a ratio of 1 to 10. Finally, 0.18 mL of the seed solution was added to the growth solution. The final solution was kept for at least 4 h at 32 °C. The solution was then centrifuged at 12,000 revolutions per minute for 10 min, reaching a final concentration of 20 mg/mL. The AuNRs exhibited strong absorbance at approximately 808 nm.

### Fabrication of CS/CS-AuNRs hydrogel

Three hundred microliter of CS (Sigma-Aldrich) were dissolved in 1.14 mL of 1 N NaOH under vigorous stirring. The cross-linker EGDGE (Sigma-Aldrich) was then added, 300 μL (21%), and the solutions were mixed thoroughly. In order to fabricate a CS-AuNR hydrogel, the solution was mixed with concentrated AuNRs to two final concentrations, 20 mg/mL and 40 mg/mL. The mixture with or without AuNRs was degassed under vacuum for 10 min, after which it was transferred to a syringe for molding. The latter was sealed and placed in room temperature (RT) for 48 h. Cross-linking of the polymer took place and a gel was formed. The cylindrical hydrogel was removed from the syringe, cut into 3 mm slices, washed in DDW in glass vials, and kept at RT for 3 days to obtain equilibrium swelling. The water was replaced daily. Washing was carried out to remove any residual polymer/cross-linker from the gel.

### Rheology

The hydrogel rheological properties were examined by Discovery HR-3 hybrid Rheometer (TA Instruments, New Castle, DE, USA) using both Peltier and upper heated plates in order to maintain temperature and 8 mm diameter parallel plate geometry. Sample evaporation was prevented by DDW drops that were set on the Peltier plate. The samples were loaded after crosslinking and washing at RT. The viscoelastic properties of the hydrogel were examined using frequency sweep, with the strain set to 0.63% and the frequency set between 0.01 and 100 Hz.

### Fabrication of macroporous scaffolds

In order to generate a porous and sponge-like structure, CS and CS-AuNRs scaffolds were generated. Pore structures and sizes were controlled by different crosslinker concentrations and freezing regimes before lyophilization. Therefore, the scaffolds were formed in the presence of 21% crosslinker. Then, the scaffolds were washed with DDW, snap frozen in liquid nitrogen or frozen at − 80 °C. After freezing, the scaffolds were lyophilized for 24 h.

### Degradation assay

The scaffolds were placed in a 24-well plate. Then, 500 µL solution of 1 U mL^−1^ collagenase type II (Worthington Biochemical Corporation, Lakewood, NJ) in Dulbecco’s Modified Eagle Medium (DMEM) were added to each well and the scaffolds were placed in a 37 °C humidified CO_2_ incubator with mild shaking. Untreated scaffolds placed under the same conditions but without collagenase supplementation served as a control. Solutions were changed every other day. Samples were removed after degradation times of 30 min, 24, 72, 120 and 168 h, frozen, lyophilized and weighed again.

### Cardiac cell isolation, seeding and viability assay

Cardiac cells were isolated according to Tel Aviv University ethical use protocols as previously described [19]. Briefly, left ventricles of 0–3-day-old neonatal Sprague–Dawley rats (Envigo Laboratories, Israel) were harvested, and cells were isolated using six cycles (30 min each at 37 °C) of enzyme digestion with collagenase type II (95 U/mL; Worthington, Lakewood, NJ) and pancreatin (0.6 mg/mL; Sigma–Aldrich) in Dulbecco’s modified Eagle Medium (DMEM, CaCl_2_·2H_2_0 (1.8 mM), KCl (5.36 mM), MgSO_4_·7H_2_O (0.81 mM), NaCl (0.1 M), NaHCO_3_ (0.44 mM), NaH_2_PO_4_ (0.9 mM)). After each round of digestion cells were centrifuged (600G, 5 min) and resuspended in culture medium composed of M-199 supplemented with 0.6 mM CuSO_4_·5H_2_O, 0.5 mM ZnSO_4_·7H_2_O, 1.5 mM vitamin B12, 500 U/mL Penicillin and 100 mg/mL streptomycin, and 0.5% (v/v) fetal bovine serum (FBS). To enrich the CMs population, cells were suspended in culture medium with 5% FBS and pre-plated twice (45 min). Cell number and viability were determined by a hemocytometer and trypan blue exclusion assay. Two million cells were seeded onto the scaffolds by adding 40 μL of the suspended cells followed by a 90 min incubation period (Humidified incubator, 37 °C, 5% CO_2_). Following, cell constructs were supplemented with culture medium (5% FBS) and further incubated. Cell viability was determined using a Live/Dead fluorescent staining with fluorescein diacetate (Sigma-Aldrich, 7 µg/mL) and propidium Iodide (Sigma-Aldrich, 5 µg/mL) for 10 min at 37 °C. The number of live and dead cells was determined by manual counting using NIS Elements software (Nikon) from at least 3 different microscopic field (n ≥ 3 in each experiment), visualized by inverted fluorescence microscope (Nikon Eclipse TI). Presto Blue cell viability assay (Life Technologies, NY) was performed. Samples were incubated in a media containing 10% PrestoBlue solution for 6 h, after which a sample was taken and absorbance at 570 and 600 nm was measured using a Biotek Synergy plate reader (Biotek, Winooski, VT).

### Controlled release

All release experiments were conducted in phosphate buffered saline (PBS) in a physiological pH, using a custom-made release chamber at RT. The scaffolds were placed in 0.0007 M of a solution of the protein in PBS for 24 h. The protein-loaded scaffolds were removed from the loading medium, blotted dry, and rinsed with a 1:10 PBS buffer three times every 2 h. Electrical stimulation was applied for the first time after an hour and then every 3 h using a BioLogic potentiostat (SP-150). The electrical stimulation generated an electric field between the electrode on which the scaffold was placed and one of the ground electrodes. Protein release was monitored by removing samples of the medium before and after electrical stimulation. The samples were replaced by adding an equal volume of buffer to the release chamber. The passive release experiments were conducted in the same way without stimulation.

### Release and quantification of the released factors

For lysozyme, electrical stimuli were applied using 1 V and lysozyme release was quantified by absorbance at 280 nm using Infinite M200 PRO multimode microplate reader from Tecan. For SDF-1, electrical stimuli were applied using 1 V and SDF-1 was detected using a human CXCL12/SDF-1 Enzyme-Linked Immunosorbent Assay (ELISA) kit (R&D systems, Minneapolis, MN).

### Migration assay

Jurkat cells, expressing CXCR4 receptors were serum starved in RPMI medium (RPMI 1640, Biological Industries, Kibbutz Beit-Haemek, Israel) for 4 h. Transwell plate membranes (Corning Life Sciences, Tewksbury, MA) were coated with fibronectin (Biological Industries, Kibbutz Beit-Haemek, Israel) for 1 h in 37 degrees. Three hundred and fifty μL of medium with or without the released SDF-1 were added to the bottom chambers of the transwell plates. Three hundred μL of medium containing 1.5 × 10^5^ cells were added to the top chamber and allowed to migrate for 2.5 h in 37 °C, 5% CO2. Medium from the bottom chamber was collected and cell numbers were counted by a hemocytometer (n ≥ 5 in each group).

### In vivo studies

All animal experiments were performed according to Tel Aviv University ethical use protocols. Recipient SD male rats (200–240 g, Envigo Laboratories, Israel) were anesthetized using a combination of Ketamine (40 mg/kg) and Xylazine (10 mg/kg). Subcutaneous implantation of samples was performed by creating a small incision to the back and the scaffolds were inserted into the cavity. The electrodes were connected to a stimulus generator (STG-4002, Multichannel systems), and a voltage of 2 V was applied for 2 min once a day. Eight days after transplantation the rats were sacrificed and the samples were extracted, fixed in 4% formaldehyde, and embedded in optimal cutting temperature (OCT) compound. Using a cryotome™ FSE (Thermo scientific), 12 μm thick sections were prepared and affixed to X-tra® adhesive glass slides (Leica Biosystems, Wetzler, Germany). The fixed samples were washed three times in 1:10 PBS to extract the OCT compound. The samples were incubated with primary α-smooth muscle actin antibody (1:200, Sigma–Aldrich), washed three times and incubated for 1 h with Alexa Fluor 647 conjugated goat anti-mouse antibody (1:500; Jackson, West Grove, PA). For nuclei detection, the cells were incubated for 3 min with Hoechst 33,258 (1:1000; Sigma) and washed three times. Samples were visualized using a scanning laser confocal microscope (Nikon Eclipse Ni).

### Statistical analysis

All statistical analyses were performed using GraphPad Prism 8.00 (GraphPad Software, Inc., USA). Data are shown as mean ± SEM (Standard Error of the Mean). Data were analyzed using Student's t-test. The values were considered significantly different at p < 0.05. n ≥ 3 in each experiment.

## Data Availability

All data used to support the findings of this study are available from the corresponding author upon request.
